# Lesion-symptom mapping of a complex figure copy task: A large-scale PCA study of the BCoS trial

**DOI:** 10.1016/j.nicl.2016.04.007

**Published:** 2016-04-18

**Authors:** Haobo Chen, Xiaoping Pan, Johnny King Lam Lau, Wai-Ling Bickerton, Boddana Pradeep, Maliheh Taheri, Glyn Humphreys, Pia Rotshtein

**Affiliations:** a Department of Neurology, Guangzhou First People's Hospital, Guangzhou Medical University, Guangzhou 510000, PR China; b School of Psychology, University of Birmingham, Birmingham, B15 2TT, UK; c Consultant in Old Age Psychiatry, Avon & Wiltshire NHS Trust, Green Lane Hospital, Devizes, Wiltshire SN105 DS, UK; d Department of Experimental Psychology, University of Oxford, Oxford OX1 3UD, UK

**Keywords:** Apraxia, VBM, Visuo-motor transformation, Drawing, High-level motor control, CT, Stroke, Constructional apraxia

## Abstract

Complex figure copying is a commonly used neuropsychological test. Here we explored the neural basis of the factors underlying complex figure copying (CFC), using data from the Birmingham Cognitive Screen (BCoS) in a large group of sub-acute, ischemic stroke patients (239). We computed two analyses: in the first we assessed the contribution of co-morbid deficits (i.e. in gesture processing, object use, visual neglect, pictures naming and sustained attention) to the lesions associated with CFC. In a second analysis a Principle Component Analysis (PCA) was used to isolate different underlying task components and to link to clinical neuroimaging scans. A voxel-based morphometry (VBM) analysis showed that poor CFC performance was associated with lesions to bi-lateral thalamus, lingual, right fusiform and right inferior parietal cortices (rIPC). The latter association with the posterior parietal cortex was diminished after controlling for neglect. Follow up analysis showed the neglect partially mediated the correlation of CFC and rIPC. The PCA revealed three main underlying components: (1) a component associated with high-level motor control common to different measures of apraxia and linked to the left postcentral gyrus, the right thalamus and middle frontal gyrus; (2) a visuo-motor transformation component unique to the CFC and associated with lesions to the posterior occipital and sensory cortices; (3) a component associated with multistep object use tasks which was correlated with lesions to the left inferior frontal orbital gyrus, the right fusiform and cerebellum. Using clinical symptoms, cognitive profiles and lesion mapping we showed that beyond visual perception, CFC performance is supported by three functional networks: one for high-level motor control, a visuo-motor transformation component, and multistep object use network.

## Highlights

• Symptoms comorbidity, PCA and VBM were combined to report the neuro-cognitive mechansims underlying complex figure copy.
• Beyond vision, CFC is supported by 3 functional networks: high level motor, visuo-motor transformation and object use.
• The association between right inferior parietal cortices and CFC is partially mediated by neglect.

## Graphical abstract

**Figure:**
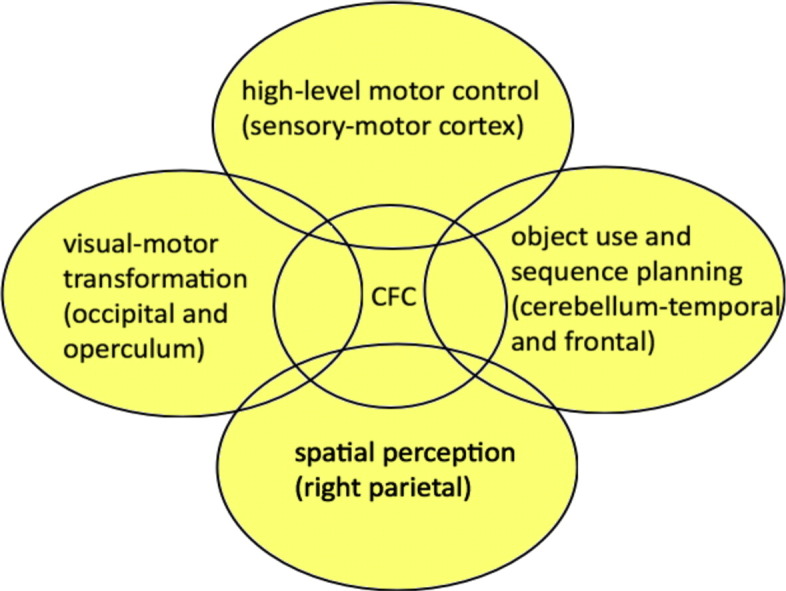

## 1 Introduction

**Fig. 1:**
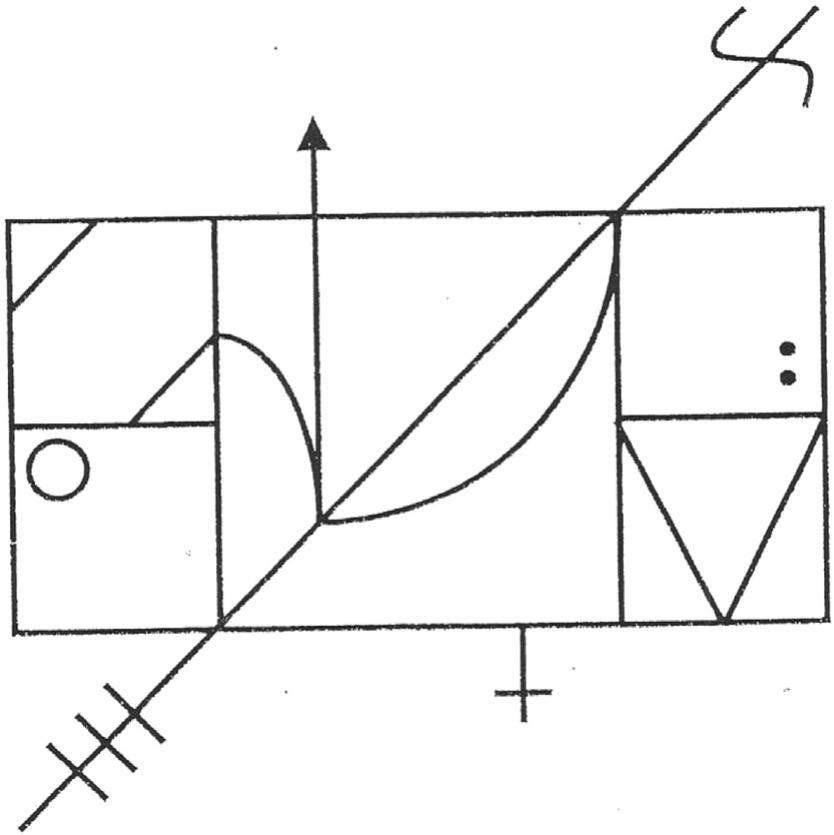
CFC task in BCoS The figure in BCoS contains a middle structure and additional structures to the left and right. There are in total 16 features. Each feature is scored on 3 criteria: presence, shape and placement (except for the Middle Square which consists the former 2 criteria). The final score is the sum of the accurate reproductions of features, achieved with a maximum of 47.

Complex figure copying (CFC), involving stimuli as the Rey-Osterrieth Figure (Rey, 1941), is a widely used clinical test. In these tasks participants are asked to copy a figure (e.g. Fig. 1), with the figure either left in front of them or removed to load visual memory. Poor performance may reflect a number of different cognitive functions including visuo-constructional ability, visual memory, executive functions (Shin et al., 2006, Watanabe et al., 2005) and processes associated with eye-hand coordination (Tchalenko and Chris Miall, 2009). To date function-lesion mapping studies have focused primarily on the processing supporting the visual and attentional aspects of complex figure copy (Chechlacz et al., 2014, Possin et al., 2011). These studies are based on specific drawing errors (Chechlacz et al., 2014) or correlates with other spatial and executive attention tasks (Possin et al., 2011). In the studies lesions to right hemisphere structures associated with visual neglect are found also to impair CFC. In the current study we focus on the cognitive processes supporting high-level motor control, visuo-motor transformations, and multi-step action, by combining CFC performance with other high-level motor tasks. In addition we used symptom co-morbidity to identify the functional role of various networks associated with CFC.

Function-lesion mapping studies indicate that both left and right hemisphere lesions contribute to deficits in drawing complex figures (Guérin et al., 1999). For example consider the postmortem structure-function study of Nielson and colleagues (Nielson et al., 1996), who examined the association of each lobe (occipital, parietal, frontal and temporal) with figure copying in Alzheimer patients. Neural degeneration in the bilateral occipital lobe, best predicted CFC performance. Similarly in studies using PET with Alzheimer patients (Melrose et al., 2013), deficits in performance in CFC tasks were associated with decreased metabolism in bilateral occipital cortices, plus also bilateral temporal–parietal regions and the right frontal lobe.

More recent studies have attempted to identify the roles of specific brain areas in drawing complex figures (Possin et al., 2011, Chechlacz et al., 2014, Biesbroek et al., 2014). Focusing on the right hemisphere, Possin et al. (2011) tested the neural degeneration that correlated with the ability to copy a figure in fronto-temporal dementia (FTD) as well as patients with Alzheimer's disease. Cortical degeneration was assessed in different lobes. They reported that right parietal damage predicted CFC performance in Alzheimer patients and the extent of damage to right middle frontal gyrus (MFG) predicted CFC performance of FTD patients. Using additional tasks the authors dissociated the functional role of the parietal and MFG. Specifically they suggested that poor visuo-spatial perception is associated with degeneration in right parietal cortex. In contrast atrophy to the right MFG correlated with deficits in spatial planning and visual working memory. However as this study focused on pre-determined region of interests in the right hemisphere, it is difficult to infer the contribution of other regions to CFC.

A different approach to isolate unique cognitive processes underlying CFC was used by Biesbroek et al. (2014). The authors compared lesion associated with impairment in CFC to those associated with impairment in judgment of line orientation (Benton et al., 1978). The sample included stroke patients who during the test showed no signs of hemianopia, visual neglect and hemiparesis for the dominant hand. Lesions were manually delineated from different types of imaging (CT, MRI) and different types of sequences (MR-T1, MR-flair). Only lesions to the right hemisphere showed reliable associations with both tasks. Specifically, lesions to a large frontal-inferior parietal network extending to superior temporal lobe were correlated with impairment in both tasks. Involvement of these regions potentially reflects visual processing and selective attention. Lesions in the right superior parietal lobe, angular gyrus and middle occipital gyrus were associated reliably with poor performance on the Rey-Osterrieth Figure copy task and not on the orientation task (Biesbroek et al., 2014). However, performances on the two tasks were not directly contrasted, which precluded direct inference on function-lesion dissociations. Using similar function-lesion mapping method, Tranel et al. (2008) tested the neuroanatomical correlate of Clock Drawing Test (CDT) with focal brain damage. The authors delineated lesion affecting two types of visual-spatial errors: lesions in right parietal cortices (supramarginal gyrus) were associated with increase in shape errors; while the lesion to left inferior frontal-parietal opercular cortices lead to increase in ‘arm’ position errors (Tranel et al., 2008).

Chechlacz and colleagues used whole brain voxel based morphometry (VBM) with stroke patients focusing on specific visuo-spatial deficits interfering with CFC (Chechlacz et al., 2014). The authors looked at the type of errors generated by the patients when copying a complex figure. They reported that lesions to the right thalamus and basal ganglia were associated with overall impairment in CFC. Lesions to right inferior parietal lobule and right middle frontal gyrus were associated with the amount of detail missed on the contra-lateral (left) side, potentially reflecting visuo-spatial biases typically observed in egocentric neglect. Misplacements of elements in the figure were associated with lesions to the early visual cortex and the insula. Lesions to these latter regions also impaired the ability to copy small elements in the figure, suggesting a problem with local feature processing. Lesions to the right middle temporal gyrus, on the other hand, were associated with the inability to reproduce large elements, consistent with a deficit in global processing (Chechlacz et al., 2014).

Taken together these studies highlight the multi-faceted neural processing required by the CFC task. Lesion-symptom mapping studies (Possin et al., 2011, Chechlacz et al., 2014, Biesbroek et al., 2014) have emphasized the important role of right parietal, middle frontal and middle occipital cortices in visuo-spatial aspects of CFC — either mediating spatial attention particularly on the contra-lesional side (Chechlacz et al., 2014, Biesbroek et al., 2014) or spatial planning (Possin et al., 2011). However, the role of other factors such as high-level motor functions and the transformation of visuo-to-motor representations remains unclear and is debated (Gross and Grossman, 2008).

Visuo-motor transformation is hypothesized to involve two main steps, visual perception and eye-hand coordination (Sanghavi and Kelkar, 2005). Eye-hand coordination has been studied at different levels including object manipulation tasks (Johansson et al., 2001), target reaching actions (Carey et al., 2002), and visually guided tracing and drawing/copying (Gowen and Miall, 2006, Gowen and Miall, 2007, Ogawa and Inui, 2009). Deficits in target reaching actions may be seen in optic ataxia patients (Battaglia-Mayer and Caminiti, 2002), and are frequently associated with lesions in the left superior parietal lobule (Auerbach and Alexander, 1981). Deficits in eye-hand coordination may lead to tracing and drawing difficulty typically associated with constructional apraxia (CA) (Ferber et al., 2007, Guérin et al., 1999). Given that co-ordination is most frequently required with the patient's right hand, and may be mediated by the left hemisphere, then the previous emphasis on right hemisphere processes may fail to address co-ordination problems.

The neuro-cognitive processes supporting eye-hand coordination in pencil-paper tasks such as CFC has previously been investigated using functional imaging (Gowen and Miall, 2007). Participants were required to either ‘draw’ with their finger a simple geometric shape (based on a verbal probe) or trace the lines of these shapes. Regions activated when drawing or tracing a figure included the cerebellar vermis, an area surrounding the left central sulcus including the pre and post central gyri, the superior medial frontal cortex and the right precuneus and superior parietal cortex. These cortical regions along with the inferior and superior occipital and right cerebellum showed stronger response when the task required drawings as opposed to simply tracing a line. Another fMRI study asked healthy participants to copy or trace a figure using a computer mouse (Ogawa and Inui, 2009). Copying requires the reproduction of the figure at a separate location. In contrast to tracing, copying a figure requires the participant to create and hold (at least for short time), an analog mental representation of the figure or parts of it. Similarly to the study reported above (Gowen and Miall, 2007), regions around the central sulcus were activated more for copying relative to tracing. In addition copying induced a larger spread of activation in the occipital cortex including bi-lateral lingual and middle occipital cortices and bilateral intraparietal sulcus. The authors suggested that these latter regions supported the generation of an analog visual representation (Ogawa and Inui, 2009). Both studies suggested that regions surrounding the left central-sulcus, potentially supported motor-sensory processes and regions in occipital and parietal cortices contribute to visuo-motor transformation. The involvement of the inferior parietal cortex in eye-hand coordination and visuo-motor transformation are also supported by physiological data (see review Colby et al., 1995) and data on the effects of transcranial magnetic stimulation (e.g. Van Donkelaar et al., 2002).

Visual motor transformation tasks involve high-level motor control (over and above basic aspects of limb control). In neuropsychology, deficits to high-level motor functions are often referred to as praxis deficits. Apraxia is defined as an inability to perform complex actions and carry out skilled motor acts despite preserved sensory and motor abilities (Gonzalez Rothi et al., 1991). The symptoms of apraxia can include a failure to process gestures, a failure to interact with objects, failures to complete sequenced daily tasks and (more arguably) also the ability to build and construct figures (Gross and Grossman, 2008). The precise relations between these different aspects of apraxia, however, are not well understood. For example, poor gesture performance is typically associated with damage to left parietal and middle frontal cortices (Koski et al., 2002) and the basal ganglia (Leiguarda and Marsden, 2000), whereas CFC performance can be disrupted after right hemisphere lesions (Chechlacz et al., 2014, Possin et al., 2011, Biesbroek et al., 2014).

Moreover, impairments in CFC are also reported to co-occur with aphasia (Perren et al., 2005, De Witte et al., 2008), spatial neglect (Linden et al., 2005), visual agnosia(Paterson and Zangwill, 1944) and sustained attention (Seidman et al., 1997). While the prevalence of these comorbidities is unknown, given the complexity of CFC, it is important to extract covering effects of these cognitive functions when investigating lesion-symptom mapping in relation to CFC. This was not done in previous studies (Possin et al., 2011, Chechlacz et al., 2014, Biesbroek et al., 2014).

In the present study, we revisited the question regarding the lesion–correlates of CFC, focusing now on how these relates to high-level motor deficits, and other cognitive co-morbidities. We used a sub-set (~ 2/3) of the patients reported in Chechlacz et al. (2014). All patients were assessed using the Birmingham Cognitive Screen (BCoS, Humphreys et al., 2012). To ensure homogeneity of lesions we included only ischemic stroke patients and patients who were originally right handed. To reduce potential effects of cognitive rehabilitation and post-stroke plasticity we included only patients tested within 1 month of the stroke. To create function-lesion mapping, the behavioral data were combined with clinical neuroimaging (CT) using VBM. We first systematically assessed the impact of potential cognitive co-morbidities on the mapping of lesion to CFC. This was done by controlling for different cognitive covariates in the general linear model. All the data was extracted from the BCoS (Humphreys et al., 2012). We specifically examined the potential neural overlaps of the following deficits with CFC: 1) high-level manual processes (assessed by gesture tasks), 2) motor sequenced task requiring interaction with object (assessed by a multi-step object task), 3) visual spatial neglect (assessed by the Apples cancellation task), 4) sustained attention (the auditory attention task), and 5) high-level visual deficits, object agnosia (assessed by a picture naming task). To assess the validity of this comorbidity we counted single cases that demonstrate overlap of deficits. We further formally compared between the different models focusing on specific region of interests (ROIs).

Our main interest in this study was to investigate processes supporting high-level motor control, visuo-motor transformation and action sequencing aspects of CFC. Therefor a follow up analysis explored the neural structures underlying CFC in relation to sensory-motor cognitive components as measured by tasks such multi-step object use, gesture production, gesture recognition and meaningless gesture imitation. Principle component analysis (PCA) was used to tease apart the various cognitive components underlying CFC performance and its relations with other praxis measures. We used VBM to identify the neural correlate of the latent variables identified by the PCA.

## 2 Method

### 2.1 Participants

The BUCS trial tested nine hundred and six patients using the BCoS battery, after being admitted to the hospitals for stroke across the West Midlands (United Kingdom) (see Bickerton et al., 2015 for details). The inclusion criteria were as follows: the patient should 1) be within 3 months of a confirmed stroke; 2) be judged by the clinical team to be able to concentrate for at least 30 min to enable the tests to be administered; 3) have sufficient command of English to follow the instructions, and 4) have given written consent to participate. The study was approved by the National and local NHS ethical committees.

In this paper, we first excluded patients with hemorrhagic lesions (*N* = 43), patients who were left-handed (*N* = 76), and patients not assessed on the CFC due to fatigue or other reasons (*N* = 123). Furthermore there was exclusion of patients for whom all BCoS assessments took place more than one month post-stroke (*N* = 209) or had CT scans taken more than one month post-stroke (*N* = 155). This was done to increase homogeneity and reduced potential effects of rehabilitation.

Finally, in order to prevent artifacts in the neuroimaging analyses, we removed patients who either did not have a CT scan, or had enlarged ventricles or poor quality CT scans (*N* = 61). Our final sample included a total of 239 ischemic stroke patients (Chechlacz et al., 2014 included 358 patients, including left handed, hemorrhage and > 1 month post-stroke).

The study sample comprised 103 males and 136 females. The average age was 70.67 ± 12.88 years, and the average years of education was 12.50 ± 2.87. Table 1 shows the demographic and clinical data for the patients.

The patients were assessed in a quiet room within the hospital. At the time of testing the patients and the examiner were blind to the area affected by the stroke.

### 2.2 Behavioral measures

#### 2.2.1 Cognitive profile

We assessed the patients' cognitive profile using the BCoS battery (Humphreys et al., 2012). BCoS is a cognitive screening instrument that assesses performance across a broad range of cognitive abilities. It takes about 1 h to administer and generates cognitive profiles of individuals within 5 cognitive domains: (1) Attention and executive functions, (2) Language, (3) Memory, (4) Number Skills and (5) Action planning and control (Praxis). Importantly, the test is designed to maximize inclusion for stroke patients whilst generating test results that are less biased by the co-occurrence of language or spatial attention problems, which can otherwise have a co-varying impact on performance (e.g., avoiding contamination by aphasia and neglect by using forced-choice tests and vertical layouts).

#### 2.2.2 Complex figure copy

Patients were asked to copy a complex figure (CFC, Fig. 1) as accurately as possible. The figure in BCoS contains a middle structure and additional structures to the left and right. There are in total 16 features. Each feature is scored on 3 criteria: presence, shape and placement (except for the Middle Square which consists of the former 2 criteria). The final score is the sum of the accurate reproductions of features, achieved with a maximum of 47. Cutoff scores were derived from 100 control participants without a history of brain lesion. Participants who achieved an overall score of < 42 points (age group of < 64 years), 41 points (age group of 65–74 years), and 37 points (age group of > 75 years) were classified as impaired in this task.

#### 2.2.3 High-level motor covariates (praxis)

Four praxis tasks used to assess other high-level motor function, described in detail in Bickerton et al. (2012).

In *the Multi-Step Object Use Test* (*MOT*) the patient is required to perform a sequence of actions with two objects (a battery and a torch, presented along with distractors) to carry out an instruction (light the torch). The task assesses patients' ability to select the correct object and follow a series of actions in order to achieve a goal. It also tests the ability to manipulate the objects and to position them in correct spatial orientations as the objects are employed. Scoring discounts problems due to primary motor deficits.

In the *Gesture* Production (GP) task, the patient has to demonstrate six gestures based on verbal commands, 3 were transitive (e.g. ‘combing hair’) and 3 intransitive (e.g. ‘hello’).

In the *Gesture Recognition* (*GR*) test the patient has to recognize 6 gestures demonstrated by the examiner.

In the *Meaningless Gesture Imitation* (*MI*) test the task was to mimic four meaningless gestures with the less affected hand.

#### 2.2.4 Non-praxis covariates

*Orientation*: the patient was asked to reply to 8 open verbal questions to test access to personal information.

*Comprehension*: a rating score based on the clinical judgment of the examiner, concerning language comprehension during the whole assessment.

*Egocentric Neglect*: a measure of spatial attention biases, assessed primarily by the spatial asymmetry score on the Apple cancellation test (Humphreys et al., 2012) (205 patients) but in some cases by a key cancellation task (cross all the keys on the page (85 were assessed on both) (see Bickerton et al., 2011). Egocentric neglect was measured by comparing performance for targets in the left and right visual fields. The scores on the key cancellation task were transformed to match the Apple cancellation task using linear regression estimated from 198 patients in our database who performed both tasks. The conversion formula was: egocentric neglect score in the Apple cancellation test = 0.6088*(egocentric neglect scores in key cancellation test) + 0.5078. The final scores represented the extent of the spatial attention bias, ignoring its direction.

*Picture naming assesses agnosia and language abilities* (Lau et al., 2015): Patients were required to name 14 line drawings of objects.

*Auditory attention*: the participant had to selectively detect three target words and ignore distractors, with 54 stimuli presented across 2 min.

*Barthel index*: This used ten variables describing activities of daily living and mobility. A higher number indicates a greater likelihood of being able to live at home independently (Mahoney and Barthel, 1965).

### 2.3 Neuroimaging assessment

All the patients had their CT scans acquired when they were admitted to the hospital by using Siemens Sensation 16, GE Medical System Light Speed 16 and Light Speed Plus with an in plan resolution of 0.5 × 0.5 mm and a slice thickness between 4 and 5 mm. The average days that CTs were acquired 2.8 days post-stroke, with a standard deviation of 4.85.

### 2.4 Pre-processing of brain images

The data were processed using an identical procedure to the one reported in previous studies using the same database (Chechlacz et al., 2014, Lau et al., 2015). We used SPM12 (Statistical Parametric Mapping) to preprocess the data. DICOM files were first converted to NIFTI format. Consequently, we normalized the data by transforming images into the MNI space. Following this we applied the unified segmentation algorithm (Seghier et al., 2008). The unified model is used to draw the deformable tissue probability maps (also called priori tissue class). The a-priori tissue maps indicate the probability of the voxel belonging to one of the six types of signal expected in a brain: GM (grey matter), WM (white matter), CFS (cerebrospinal fluid), bone, fat and air. As a consequence of stroke a 7th abnormal tissue type representing the lesion was also proposed to be present. To account for this we followed Seghier et al.'s (2008) approach and added an additional a-priori map. We estimated that there would be 10% probability that either GM or WM consist of abnormal tissue; the 10% was considered based on the ratio between lesions volume of the patients and the brain size from the same group (see Chechlacz et al., 2014 plus on for details). Finally, we applied a single Gaussian normal distribution to classify the intensity of the grey and white matter and two Gaussian distributions to classify for the intensity of abnormal tissue. To accommodate with the random field theory we smoothed the segmented GM and WM by using a 12 mm^3^ FHWM Gaussian Kernel.

### 2.5 Behavioral data analysis

Missing data for all covariates were replaced by the group average. The amount of missing data for each task ranged from 0% to 7.1% with an average of 1.79%. To estimate the relation between the CFC and demographic data along with all the other covariates, Pearson's correlation (two-tailed) analyses were performed. All together we computed 16 correlations, and the results were corrected for multiple comparison using Bonferroni correction.

To identify underlying cognitive components of the CFC we used a PCA analysis. Before the PCA analysis, a KMO and Bartlett's test were performed across the four praxis tasks (MOT, GP, GR, MI) and CFC. The KMO value is 0.786 (over 0.6) and significance level for the Bartlett's test (332.274) with 10 degrees of freedom is below 0.001. This result indicated that there was correlation in the data selected and the distributions of data meet the assumptions of multivariate analysis. We re-scaled the raw scores of each task linearly to range between 0 and 20 to account for the difference of the maximum scores of the five tests. A PCA analysis then was computed on the rescaled data. The PCA teased apart the differential and shared components of the CFC with the four other praxis tests. In brief, PCA aims to reveal latent variables by projecting the data onto a new space defined by the components. Each new component is a linear combination of the weighted original scores. Higher loading (weight) means larger contribution of a specific task to this component. The directional sign (±) of the loading are only meaningful when comparing the contribution of each task to the component. If all signs point to the same direction (±) this means that component reflect a shared latent variable underlying all tasks. If the signs are opposite, it means that the component dissociate the two tasks. As our main focus in on CFC, for simplicity we ensured that when reporting the loading of the CFC these are always positive, hence when needed we flipped the loading signs in the component.

### 2.6 Voxel-based morphometry (VBM)

To compute the correlation between the behavioral results of the CFC in relation to grey matter lesions, we used random effects analyses within the general linear model framework (Ashburner and Friston, 2001). We used the raw CFC scores and the components extracted from the PCA analysis in separate models. In order to reduce the potential impact that some demographic and clinical factors might have on cognitive performance and brain lesion, the following measures were included as covariates of no interest in all models: age, gender, years of education, interval between stroke and CT scanning, interval between stroke and cognitive testing, Barthel Index, ability to use the dominant hand, orientation and comprehension.

Models using the CFC raw scores: Model 1 included the CFC raw data with no additional cognitive covariates. Model 2 added the 4 praxis tests from BCoS as covariates. It included the following tests: Multi-step Object Use, Gesture Production, Gesture Recognition, and Meaningless Gesture Imitation. Model 3 added the scores for Egocentric Neglect. Mode 4 accounted for Auditory Attention and model 5 controlled as well for Picture Naming.

To formally compare the impact these models had on the lesion pattern we computed the log evidence of each model in a region of interest, using the SPM function (spm_vb_regionF.m). The difference between the log evidence was used to infer which model fit the data best (a difference larger than 3 assume sufficient evidence to support one model over another). This was computed for regions that showed different level of association with CFC as depending on the specified model (rIPC, aCG, see results), as well as on a region that was not affected by the models (rFFG). For these analyses we extracted the probability of grey matter values in each patient from each ROI. This was represented as an eigen variate of 6 mm sphere centered around the peak. (Supp Table 1).

When the associations of the lesion and CFC was affected by the inclusion of specific covariates (rIPC, aCG), we run further analysis to establish the type of relations between the CFC, the brain region and the cognitive covariate. This was done using structural equation modeling implemented in SPSS-AMOS. We used the difference between AIC to compare between the models and infer the relation pattern. Detailed of compared model and results are presented in the supplementary materials (Supp. Table 2).

Finally we designed a model that included all the PCA components but did not include the cognitive covariates above. The analysis of the PCA-VBM focused on components that are most clearly and meaningfully (CFC loading > 0.4, and explained > 10% variability) linked to latent variables associated with variability in CFC.

We focus on results that survived cluster level family wise error correction with a voxel reliability of *p* < 0.005 uncorrected. This was done due to the nature of the data and the expected result pattern. The data was segmented grey matter images of patients with relative large lesions. These images were smoothed to 12 × 12 × 12 FWHM, as recommended for VBM to adhere with the continuity assumption of the random field theory. Given this data we anticipated that behavior would correlate with relative large lesions (cluster size), rather than with focal peaks. The choice of *p* < 0.005, uncorrected at the voxel level, was done as software typically relying on cluster level correction, tend to use more lenient voxel threshold. For example FSL, which relies on cluster threshold correction and by default, uses *p* < 0.01 uncorrected for the voxel threshold. For completeness we report in the tables all clusters that had > 150 voxels, this is equivalent to *p* < 0.003 uncorrected at cluster level; expected number of voxels by chance per cluster was 14.

## 3 Results

### 3.1 Behavioral results

#### 3.1.1 Complex figure copy

**Fig. 2:**
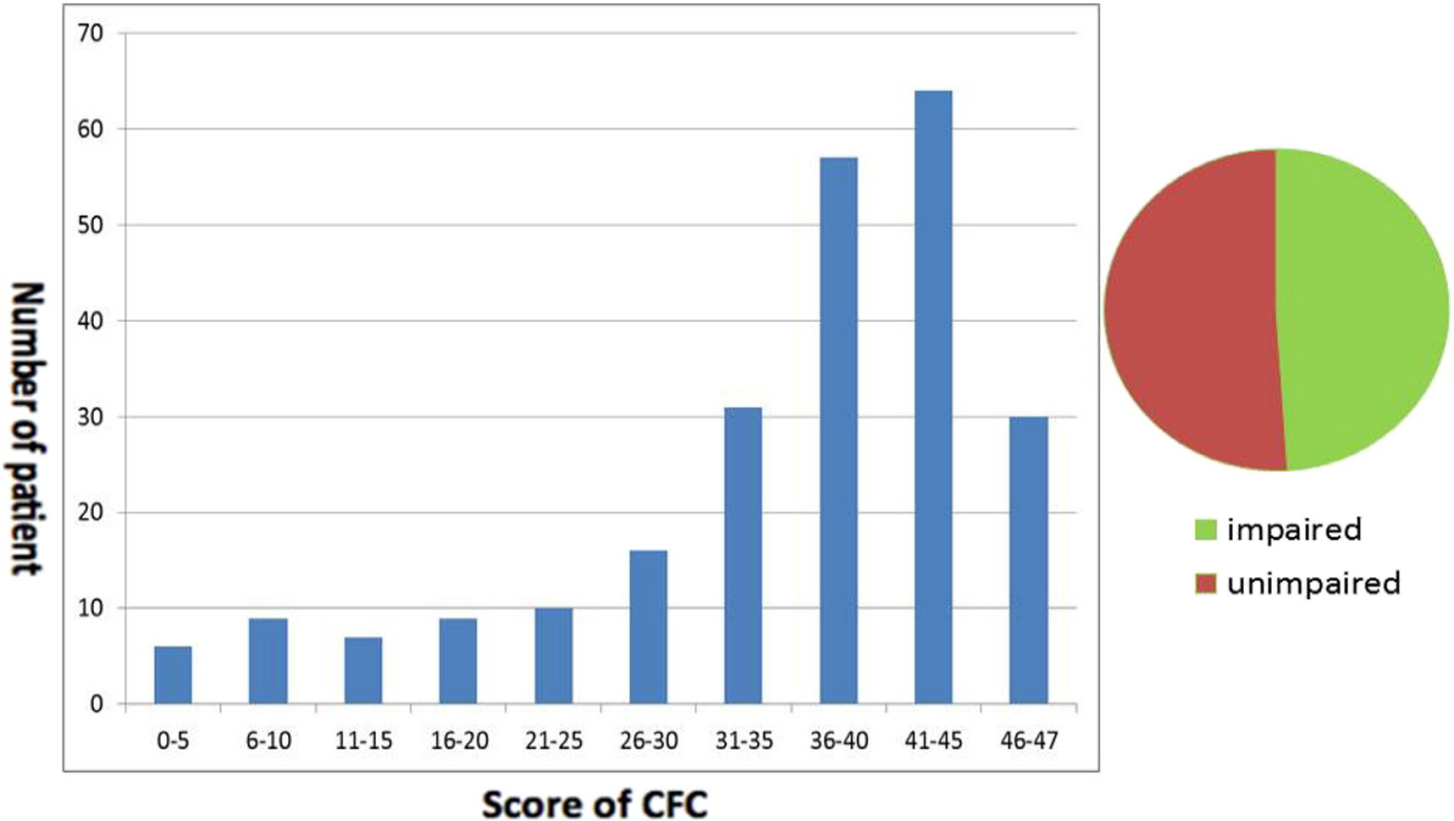
The distribution of performance in the CFC task. Participants who achieved an overall score of < 42 points (age group of < 64 years), 41 points (age group of 65–74 years), and 37 points (age group of > 75 years) were classified as impaired in this task. About half of the patients got impaired in CFC test.

The patients recruited in our study had an average score of 35.20 (SD: 11.30) in the CFC task; performance varied with 14 patients scoring < 10, and 94 scoring higher than 40 (see Fig. 2, for the distribution). Compared to the cut-off points established from the age-matched healthy controls (Humphreys et al., 2012), 117 were classified as impaired.

#### 3.1.2 Correlation of CFC with the demographic data

The correlation results are reported in Table 1. Gender and education did not affect performance on the CFC. Age had a weak negative impact, with older individuals preforming worse than younger ones. The date from the stroke to the test was also weakly negatively correlated with CFC impairments. This may reflect a sampling bias, in which the more severe patients are likely to be assessed in the rehabilitation wards at a later time point after the stroke. As expected, the Barthel index and task comprehension had significant weak correlations with CFC, indicating that patients with worse performance in activities of daily living and worse understanding were likely to have a lower score in the CFC test. There were no significant correlations between CFC and whether the patient copied figure using their dominant hand or not.

#### 3.1.3 Correlation of CFC with performance in other cognitive domains

Not surprisingly in the current study, CFC showed positive weak to moderate correlations with all the four praxis tasks: MOT, GP, MI and GR.

CFC also correlated with visual spatial neglect. Patients who had page-based asymmetrical spatial attention (ego-centric neglect) were poorer at copying the complex figure (see Table 1), consistent with neglect impacting on performance on the CFC test. Finally, CFC also correlated with orientation, sustained attention and picture naming. These results demonstrated the prevalence of comorbid cognitive deficits in stroke patients (see also Bickerton et al., 2015). In order to describe in more detail the prevalence of comorbidities in our sample, we computed how many patients who were classified as impaired in CFC were also impaired in other cognitive domains. Of the 117 patients who showed a deficit in performing the CFC, the most common (56%) comorbidity was sustained attention assessed by the auditory attention task (Humphreys et al., 2012). In addition 43% were also impaired in picture naming, suggesting the presence of object agnosia or aphasia. Visual neglect was observed in 39.3% of the patients impaired on CFC. In relation to the other apraxia tests, 39.9% of the patients were impaired at imitating meaningless gestures, 33.3% failed the multi-object use task, 26.5% the gesture production and finally 22.2% the gesture recognition tasks. These relatively high comorbidities highlight the importance of controlling for potential covarying cognitive deficits in lesion-symptom mapping.

Given the potential relations between the various aspects of apraxia and poor performance on CFC, plus the relatively high correlation of the praxis tasks and CFC, we used PCA to identify the underlying cognitive components of CFC. We applied a PCA to the re-scaled raw scores of the five praxis tests to identify the shared and differential components between CFC and other praxis tests. We focused on the components that involved CFC.

Three components, involving the CFC explained 86% of the variability (Table 2). As PCA is a data driven approach, interpretation of the component's meaning is speculative to a degree. Here we offered one possible interpretation but discussed alternatives interpretations of the components in the discussion. The first component was shared among all the 5 tests (all the tasks loading ranged from absolutely value of 0.34 to 0.56) and explained 53% of the variability. The least contributing variable was the gesture recognition task. We assumed that this component represented high-level motor control, required by all praxis tasks. The second component differentiated primarily the CFC from the MOT. This component explained 18% of the variability. We assumed that component 2 represented the cognitive process correlated to visuo-motor transformation. Finally, the third component was representative of the shared process underlying CFC and MOT and accounted for 15% of variability. These two tasks required interaction with objects and planning of sequence actions, which suggests that component 3 represents interacting with objects and action planning. We note that component 4, is loaded primarily on the gesture recognition task (0.61) but also on CFC (0.34), dissociating both from meaningless imitation (− 0.71). This component explained only 8% of the variability in the data. We did not included component 4 in any further analysis, as we believe it primarily represent dissociation between the two gesture tasks. In addition the contribution of CFC was smaller than our threshold, as well as the amount of variability explained by this component.

In a supplementary analysis we included the other cognitive tests picture naming, neglect, auditory attention in the PCA (Supp Table 3). The first two components were similar to the ones observed when including only the praxis tests. While the third component, which linked CFC and MOT and differentiated them from the other tasks, also loaded on neglect. This suggests that cognitive mechanism underlying this third component may also support visual attention processing. Taken together when considering the 2nd component dissociating CFC from MOT in both PCA analyses; we suggest that this component is more likely to reflect visual motor transformation processes rather than visual-spatial processing (as neglect was loaded on the 3rd component).

### 3.2 Neuroimaging results

We related the behavioral measures to the neuroimaging data to explore the lesion-symptom correlates with the CFC.

#### 3.2.1 VBM based on raw scores of the CFC

**Fig. 3:**
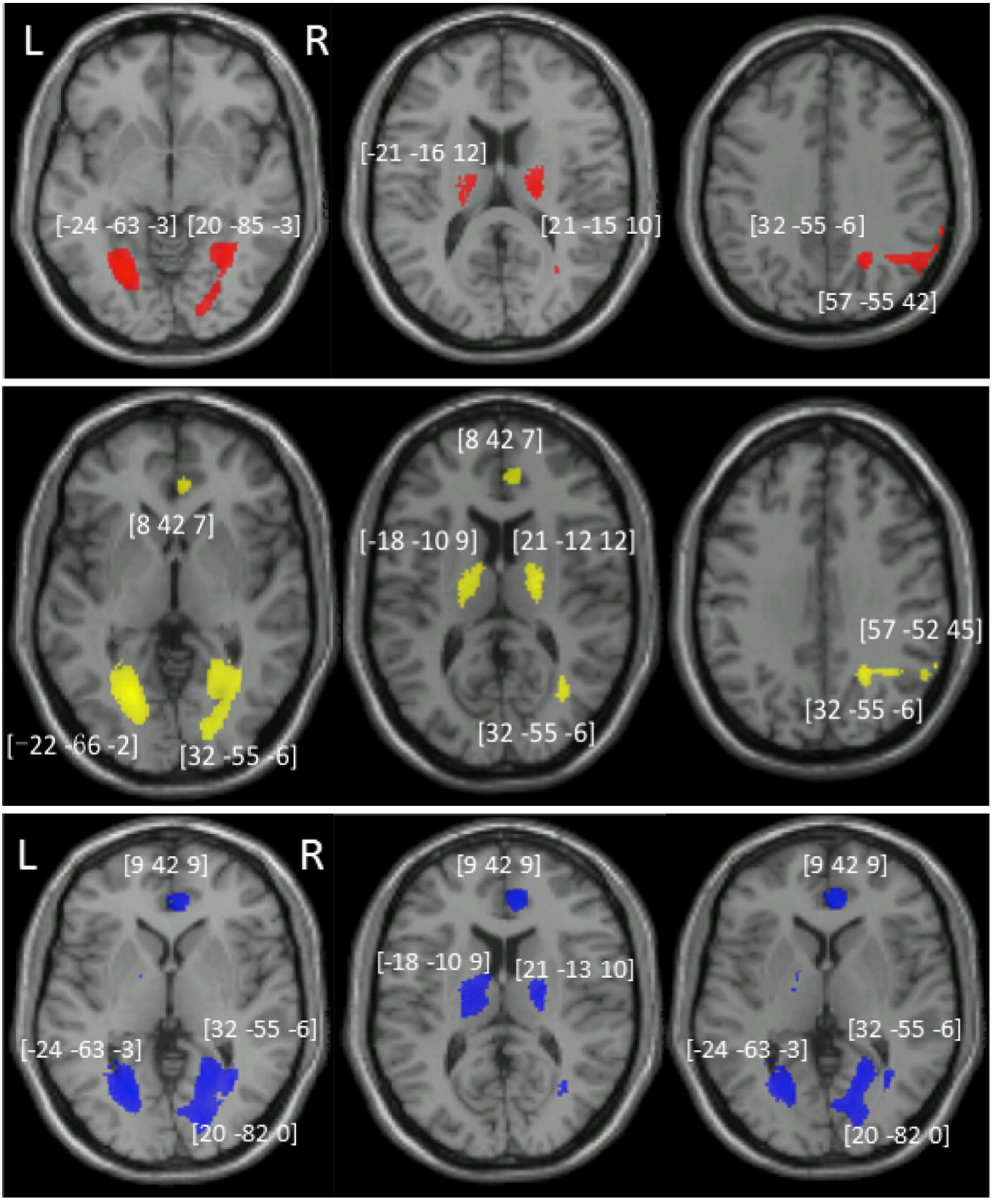
The VBM results on the CFC and after controlled for other correlated tests. VBM results showing voxels corresponding to grey matter damage in (red) CFC only, (yellow) after controlling for the other four praxis tasks, (blue) after controlling for the praxis task, egocentric neglect, auditory attention and picture naming task. The function-lesion maps are overlaid on axial T1-weighted MRI slices of the single subject canonical template provided by SPM. The numbers in brackets represent the peak of the clusters given in MNI coordinates. Notice that lesion in aCG were reliably associated with CFC impairment only after we added the four praxis tests as covariates; while lesion to right IPG became unreliable after we added egocentric neglect as covariate. (For interpretation of the references to color in this figure legend, the reader is referred to the web version of this article.)

VBM analysis results are reported in Table 3 and Fig. 3. Based on the raw scores of the CFC, with no additional cognitive covariates, there was a significant positive relationship between performance and voxels in the right inferior parietal lobe, right fusiform, bilateral lingual gyrus, and bilateral thalamus (model 1). The significant correlations between worse performance in CFC and lesions in bilateral thalamus, bilateral lingual gyrus and right fusiform were observed even after we controlled for the other four praxis tests (model 2), egocentric neglect (model 3), verbal working memory, selection and sustained attention (the auditory attention test from BCoS) (model 4) and picture naming (model 5). Interestingly, after including the praxis covariates (models 2–5) voxels in the right anterior cingulate gyrus also correlated with CFC performance. In summary, the results suggest that bilateral thalamus, lingual, right anterior cingulate and right fusiform lead to an impaired ability to copy a complex figure even after controlling for various cognitive co-morbidities.

**Model comparison using log evidence** - We formally compared between the five models in three regions of interests (ROIs) using log evidence. 1) The fusiform gyrus was least affected by the changes in the model covariates. For this region the best model was the 3rd model where we included the four praxis tasks and the neglect scores. 2) The right IPC that showed below threshold association with CFC once we added the neglect covariate. For this region the best model was also the 3rd one which included praxis and neglect; 3) The aCG which showed above threshold association with CFC after we controlled for the praxis tasks. Here the best model was the 1st one, where there was no control for any cognitive tasks (apart from orientation). These results demonstrate that the best-fitted model varies depending on the region selected and there is no one correct answer that fits all. (Supp Table 2).

**Comparing the relations between the CFC, ROI and cognitive covariates** - We used SEM to investigate in more details the relations between CFC, neglect and right IPC. We used the AIC values to select the best fitting model, which take into account the fitting accuracy and model complexity (number of parameters). We established three different models to describe the relations between the three variables: 1) rIPC independently supports neglect and CFC, 2) rIPC involvement in CFC is fully mediated by neglect, and 3) rIPC involvement in CFC is partially mediated by neglect. Based on the value of AIC, the analysis suggested that the best model is the 3rd one, in which the correlation of rIPC and CFC are partially mediated via neglect.

A similar analysis was performed to explore the relation between aCG, CFC and the praxis tasks. The result indicated that the best model was that aCG and Praxis tasks independently explain CFC performances. In other words, variability in CFC that cannot be accounted by praxis deficits was associated to aCG lesion. (see more details in Supp Table 2).

#### 3.2.2 VBM based on PCA scores for complex figure copy

**Fig. 4:**
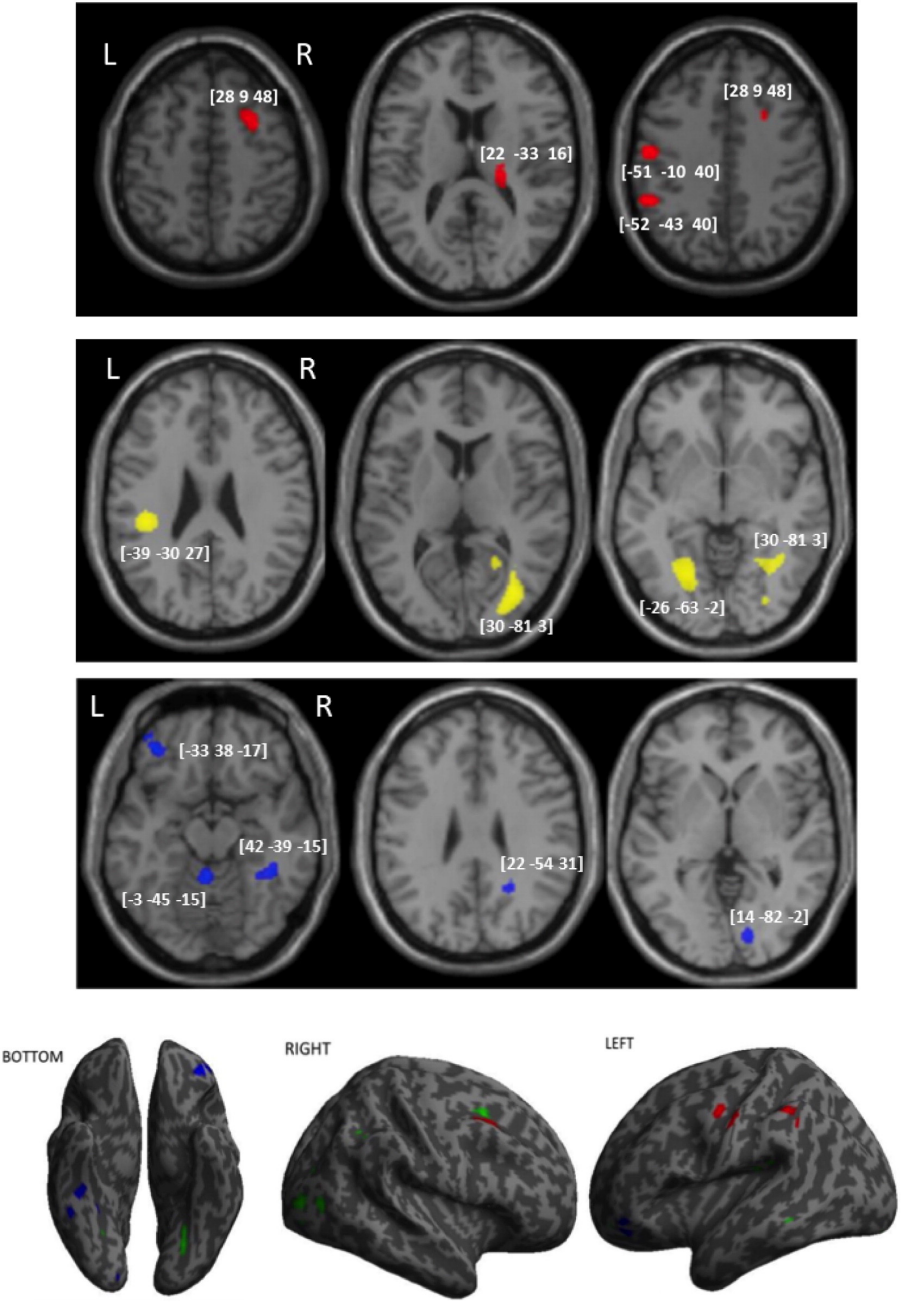
VBM analysis based on the first three components from PCA result. VBM results showing voxels corresponding to grey matter damage in (red) the first shared component refereeing to high-level control, (yellow) the second component indicating visual-motor transformation, and (blue) the third component representing interacting with objects and planning in multi-step task. The function-lesion maps are overlaid on axial T1-weighted MRI slices of the single subject canonical template provided by SPM. The numbers in brackets represent the peak of the clusters given in MNI coordinates. (For interpretation of the references to color in this figure legend, the reader is referred to the web version of this article.)

These results are presented in Table 4 and Fig. 4. The analysis focused on the first three components (PC1, PC2, PC3), each explained > 10% of the variability in the data. As the main aim of the current study was to map lesion associated with CFC, we selected components that had clear and meaningful association with CFC. We further focused on contrasts linked to latent variables in CFC. In other words, we mapped lesions that predict poorer performances of CFC.

Based on the loading pattern of the first shared component, we suggest that it represents high-level motor control. These were associated with reduced density in GM of the right thalamus, the right medial frontal cortex, and the left postcentral gyri. The second component, we identified with visuo-motor transformation, poorer CFC performances on this component were associated with reduced GM in the right lateral occipital, right fusiform, left lingual and right rolandic operculum gyri within the inferior parietal lobe. Finally, we observed that the third component associated with object interactions/neglect linked to lesions to the left inferior frontal orbital, right fusiform and cerebellum.

A supplementary analysis (Supp Fig. 1, Supp Table 4) included the PCA components that were derived from all the eight cognitive tasks. The lesions associated with the shared (PCA1) and the drawing (PCA2) components were similar to the one reported above — though the right thalamus was no longer reliably associated with shared deficits across tasks. More interestingly, as component 3 was now loaded on the CFC, MOT and neglect, it was primarily associated with lesions to the right inferior parietal cortices, suggesting that this component reflecting spatial attention and not only being unique to the visuo-motor transformations associated with drawing.

### 4 Discussion

The aim of the current study was to reveal the underlying cognitive-neural components associated with copying a complex figure, as routinely tested in neuropsychological batteries. We first observed high comorbidities of failure to copy a complex figure with sustained and visual attention deficits. High comorbidities were also observed with picture naming and the various praxis tasks. The behavioral results are consistent with the idea that CFC performance depends on common high-level motor coding, shared with other praxis tasks, as well as spatial attention, and assessed using visual cancellation.

The VBM results showed that CFC was associated with lesions to bi-lateral thalamus and lingual gyri, the right inferior parietal lobe and fusiform gyrus. Interestingly, after controlling for spatial neglect, the right inferior parietal lobe showed no significant correlation with CFC. Further SEM analysis showed that lesion to the rIPC correlated with deficits of neglect and CFC; with the later deficits being partly mediated by the neglect deficits. In contrast, lesions to the anterior cingulate gyrus (aCG) were associated with CFC performance after we controlled for the four praxis tests. Further SEM analysis showed that aCG explained variability in CFC that was not accounted by the four praxis tasks.

Focusing on aspects of the CFC linked to high-level motor control, we next applied PCA to dissociate underlying cognitive components that contributed to poor CFC performance. There were three main components when CFC performance was considered alongside performance on the praxis tests. The first component explained variability across the five praxis tasks, representing shared involvement of high-level motor control. Low scores in this shared component correlated with lesions to the left postcentral gyrus, the right thalamus and the middle frontal gyrus. The second component was unique to figure copy and dissociated it from the multi-step object task, suggesting that it reflects the visuo-motor transformations required specifically for drawing. Impairments in this process were associated with lesions to the right middle occipital gyrus, the left lingual gyrus and rolandic operculum. Finally, a third component linked the CFC and the multistep object tasks separate from the gesture tasks. Without spatial attention taken into account, deficits were predicted by lesions to the left inferior frontal orbital gyrus, the right fusiform and cerebellum. We discuss each of these findings separately next.

#### 4.1 Incidence and comorbidity of deficit in CFC

Within one month post an ischemic stroke, about half of the patients in our study showed impairments in the CFC task from the BCoS battery (Humphreys et al., 2012). Considering that the analysis excluded patients who were unable to concentrate for at least 30 min or had severe limb paralysis, the incidence of CFC impairment may be even higher.

The ability to copy a complex figure was found to be associated with several cognitive functions, revealed by both correlation analysis and the prevalence of comorbid impairments. Significant and positive relationships with CFC were found with the four praxis tasks, neglect, picture naming, and auditory attention tests. The relatively high-level of symptom-association may not be surprising given the multifaceted processes required for successfully copying a figure (van Sommers, 1989). The high prevalence of deficits in complex figure copy and the other praxis tasks was also evident in the PCA analysis which revealed that most of the variability in patients' performance could be explained by a single shared component. We interpreted this component to reflect high-level motor control. As it was more weighted on the tasks that involved manual action compared with the gesture recognition task. However, we cannot role out the possibility that this component also reflect stroke severity. Or any other potential shared latent variables that affect all five tasks.

The PCA analysis also revealed dissociations between CFC and the other praxis task. Specifically one component dissociated CFC from the gesture and object use task that we propose reflects visuo-motor transformation processes in drawing. Could that component be related to visual-spatial processing primarily? Visuo-motor transformation is hypothesized to involve two main steps, visual perception and eye-hand coordination, including the processes of visuospatial perception. Complex figure copying is a visually guided copying task requiring not only visuospatial processes but also eye-hand coordination. Furthermore, in the supplementary analysis we included neglect (assessing visuospatial processing) in the PCA analysis. In this analysis we again observed a component that primarily dissociated CFC from MOT, with minimal contribution from neglect. This component was associated with a similar lesion map as the one in our main analysis (see below). Taken together, we therefor interpreted this component to primarily depict visual-motor transformation processing.

A third relevant component grouped CFC performance with performance on the multi-step object use tasks, distinguishing them from the gesture tasks. We suggest that this reflects interaction with objects and planning of sequenced actions. As the gesture tasks are a single action step task compared to CFC and multi-step object used which are multi-step action, and the later two tasks involving using tools (pen or torch). Taken together the findings suggested that deficits in CFC were linked to a general higher order motor deficit, but they also demonstrated an involvement of spatial-visual attention, organization and planning processes. The relation between the different praxis tasks and CFC is also manifested in the imaging data, which we described below.

The analysis of the behavioral results suggests sustained attention as potentially contributing to performance on the CFC. This is consistent with sustained attention being a basic resource for maintaining a cognitive set (van Sommers, 1989). For example, beyond planning and organization, patients need to be able to maintain their focus and concentration on the task to enable successful task completion. However, we did not observe a specific component related to shared variability of sustained attention and CFC in the PCA results (Supp Table 3). Furthermore, none of the identified clusters that associated with CFC were modulated by performance in the sustained attention task. This may reflect the fact that sustained attention underpins success in any cognitive task, and is not a unique requirement for CFC.

As predicted, visual neglect was prevalent in patients who failed the CFC task. Spatial neglect deficits may hinder CFC due to poor visual spatial scanning and spatial representation (Behrmann and Plaut, 2001, Chechlacz et al., 2014). We also observed high prevalence of comorbidity of CFC and picture naming deficits. As mentioned before, picture naming impairments are found in both aphasia and agnosia. Praxis deficits are commonly reported together with aphasia (Goldenberg and Spatt, 2009; though see Roby-Brami et al., 2012). On the other hand, no clear link between CFC and agnosia has been found (Humphreys and Riddoch, 1987), despite having visual input analysis as a common basis for both tasks. The current imaging analysis suggests that CFC relies on processing at posterior and ventral parts of occipital cortex (see below), similar to picture naming (Lau et al., 2015).

Clinical comorbidities may potentially be explained in two ways. Firstly it is possible that the different tasks rely on the same underlying cognitive processes. Secondly, it is possible that these tasks utilize different cognitive processes but the neural structures supporting them share the same vascular territory and hence ischemic stroke is likely to affect both tasks together. To test these two explanations we used two approaches. At the neural level we tested the impact of the different cognitive tests (used as covariates) on the mapping of CFC to lesion structure. We also computed a principle component analysis identifying shared and dissociated neural components of CFC and the 4 praxis tasks.

Our results suggest that the co-morbidity of gesture tasks and CFC can be explained by shared cognitive processes which support high-level motor control and are associated with lesions to frontal motor associated cortices (see Table 4). Similarly, spatial neglect and CFC both rely on intact spatial attention processing mediated via the right inferior parietal cortex (see Table 3 and Supp Table 3). Co-morbidity of the multi-step object task and CFC can be explained by the involvement of motor schemas, interaction with objects and the need for planning and attentional control in both these tasks. Finally, neither the VBM nor the PCA results were affected by the inclusion of picture naming and sustain attention in the analyses. We therefore suggest that co-morbidities of these two tasks and CFC cannot be explained by the shared underlying specific neural-cognitive mechanisms that we have identified here. In such cases the data could be driven by large lesions affecting neighboring regions, or shared cognitive components that were not identified here.

#### 4.2 Neural structures correlated with CFC

From the VBM results, we found that bilateral thalami and lingual gyri, the right inferior parietal lobe and fusiform were correlated with the CFC raw score. The correlation of the raw CFC score and the right inferior parietal lobe disappeared after we controlled for neglect. Supplementary analyses also revealed that damage to the right IPC also correlated with the shared cognitive component that associated deficits in spatial neglect and CFC. To further explore the relation between CFC, neglect and rIPC, we set up three structural equation models that differ in their relation structure. Formal model comparison revealed that the best fitting model describes the correlation of rIPC and CFC as partially mediated via neglect (see details in Supp Table 2).

This result expanded on findings reported by Biesbroek et al., 2014. The later authors report, that right inferior parietal lesion were associated with CFC deficits even after excluding patients who showed spatial neglect. Furthermore, using partially overlapping patients sample of the same dataset, Chechlacz et al. (2014) reported that lesions within right inferior parietal lobe were associated with left omission errors in CFC task (see also Tranel et al., 2008 for clock drawing task). The right inferior parietal cortex is often reported to be associated with spatial biases (Chechlacz et al., 2010, Chechlacz et al., 2012a, Chechlacz et al., 2012b, Chechlacz et al., 2014, Mort et al., 2003, Possin et al., 2011). Therefore, we suggest that damage to the right inferior lobe, associated with impairments in CFC, is partially due to spatial attention deficits, though it also directly contributes to CFC performances independently of neglect.

Interestingly, when including the four praxis tasks in the model we also observed that lesions to the aCG reliably predicted performance in CFC. To further investigate the relation between aCG, CFC and the praxis tasks, we use SEM (see above). The results indicated that the best fitting model describes aCG contribution to CFC but not to praxis, and independently praxis is associated with CFC. In other words, variability in CFC that cannot be accounted by praxis deficits was associated to aCG lesion.

This matches a previous case study reports that damage to the aCG leads to impairments in CFC (Peru et al., 2004). Lesions of the aCG are associated with impairments of executive functions, including planning a sequence of processes, which may cause worse performance in CFC (Peru et al., 2004).

We also found that reduced grey matter integrity in bilateral lingual gyri, bilateral thalamus and right fusiform was associated with poor performance in CFC even after considering deficits in picture naming, spatial and auditory attention, and praxis. This suggests that these structures specifically contribute to processes underlying figure copying that cannot be explained by deficits in other cognitive functions. The involvement of the lingual gyri (Ogawa and Inui, 2009) and ventral occipital structures (Ogawa and Inui, 2009, Gowen and Miall, 2007) is in agreement with activation foci reported for a drawing task. The lingual lesion has also been reported to be associated with misplacing local elements (Chechlacz et al., 2014) and to be involved in tasks requiring the encoding of complex visual pictures, but without drawing (Machielsen et al., 2000). Taken together our data indicate that these posterior ventral occipital cortices may support the visual analysis of the elements and their relations in complex figures.

The association between damage to the bi-lateral thalami, which are part of the basal ganglia, observed here and also in Chechlacz et al. (2014), is in agreement with previous observations of the central role of this region supporting praxis (Leiguarda and Marsden, 2000). It has been argued that the basal ganglia support action sequencing and interactions with objects (Leiguarda and Marsden, 2000). We note too that the thalamus can also modulate attentional functions more generally (Brown et al., 1997).

We next used the components identified in the PCA, to better understand the different neuro-cognitive processes associated with CFC. This component was associated with reduced density in grey matter in the right thalamus, the right middle frontal gyrus (MFG) and the left postcentral gyrus. The observation that these regions support all tasks requiring higher-level motor function is in line with previous neuropsychological reports and functional imaging studies (Leiguarda and Marsden, 2000). These further strengthen the argument that deficits in CFC associated with lesions to these regions should be viewed as a praxis problem of higher-level motor control.

The second component loaded on the CFC alone, and differentiated CFC from the MOT. This component correlated with lesions in the right middle occipital gyrus (extending to the fusiform gyrus), the left lingual gyrus and the left rolandic operculum. As mentioned above, the lingual and middle occipital gyri have previously been found to be specifically important to drawing as opposed to simple tracing of a figure (Ogawa and Inui, 2009); while the rolandic operculum has been reported to be involved in eye-hand coordination involved in drawing (Gowen and Miall, 2007). We therefore conclude that these regions support visuo-motor transformations that are specific to drawing.

The third component dissociated the CFC and MOT from the three other gesture tasks. This component was associated with lesions to the left inferior frontal orbital gyrus, right anterior fusiform and the cerebellum-vermis. The cerebellum-vermis is frequently involved in tasks that rely on tracing and drawing eye-hand coordination (Gowen and Miall, 2007). The cerebellum (Higuchi et al., 2007) and right FFG (see review Beauchamp and Martin, 2007) are assumed to form part of the tool use network. The inferior frontal gyrus has been implicated in construction tasks (such as drawing) that specifically rely on memory (Ogawa and Inui, 2009).

Previous studies (Chechlacz et al., 2014, Possin et al., 2011, Biesbroek et al., 2014) highlight the importance of visual perception process in CFC. Beyond visual perception, our data support three main neuro-cognitive networks associated with CFC. A shared motor schema network associated with lesions to frontal motor cortices and thalamus; a network linked to visuo-motor transformation in occipital and inferior parietal-sensory cortices; and processes of planning and sequential organization of action associated with cerebellum-vermis and fusiform gyrus lesions.

#### 4.3 Methodological considerations

The current study used a sub-set of the data reported by Chechlacz's study (Chechlacz et al., 2014;) and also a different analyses approach. It is therefore, worthwhile considering the impact of these changes on the observed and reported results. In the first model, using CFC raw scores Chechlacz linked CFC only to subcortical lesions within the right hemisphere. In the current study, however we observed a larger network that included the right basal ganglia and thalamus, but also highlighted the contribution of the left thalamus, bi-lateral lingual, right fusiform and right inferior parietal to CFC. The difference between these two analysis approaches is puzzling. We note that the threshold used in Chechlacz et al. (2014) was very conservative (FWE of *p* < 0.001) which potentially may have led to increase in type II error, where potentially reliable lesions failed to rich significance. Furthermore, it could be that the more homogenous patient sample used here reduced the overall variability and led to an increase in the statistical power.

Nevertheless the two studies using different analysis approaches provide complementary results. Chechalcz and colleague conducted a detailed analysis of error types primarily reflecting visuo-perceptual deficits; in contrast here the analysis used a data-driven approach and an analysis of symptom comorbidity to reveal the lesion associated with high-level motor processing, visuo-motor transformation and object use/action sequencing.

Interesting, in line with Chechlacz et al. (2014) both analyses highlighted the importance of right parietal cortex for visuo-spatial processing in CFC. Chechlacz et al. demonstrated this using an analysis of different error types while in the current study it was shown by using performance on an independent spatial attention task. Our results indicated that the correlation between rIPC and CFC are partially mediated via neglect. In contrast to Chechlacz et al.'s analysis, we showed that the increase in misses reported to be associated with the right MFG, is potentially driven by a high-level motor deficit (here derived in the analysis of the first component).

Both analyses also highlighted the importance of the lingual gyrus and ventral visual stream to CFC; Chechlacz et al., demonstrated that these regions affected the ability to correctly position a feature within the figure. In the current study these regions were associated uniquely with CFC, and associated with visuo-motor transformation. Linking the results we can suggest the process of positioning features in objects relies on visuo-motor transformation processes specific to drawing.

In this study we used PCA analysis to identify latent variables associated with complex figure copy. We note that PCA is a data driven approach. Therefore interpretation of the component's meaning is speculative to a degree. Interpretation is done based on the weighting of the tasks on the component and the assumption regarding the processes required to complete each task. Here to validate the observed components structure, we reported the number of cases showing the dissociation observed by the PCA analysis. However, we acknowledge that these interpretations should be made with cautious.

### 5 Conclusion

The current study identified dissociable networks supporting different aspects of visuo-motor performance for copying complex figures. Specifically, we identified three networks: sensory-motor cortex for high-level motor control, posterior occipital and operculum for visual-motor transformation, and cerebellum-temporal and IFG for multi-step tasks that requires an interaction with objects.

## Figures and Tables

**Table 1:** Demographic and clinical data on the patients (*n* = 239).

Demographic data			
Variables	Descriptive(mean/median or number of patients)	Standard Deviation	Correlation with CFC
Gender (M/F)	103/136	N/A	0.120
Using the dominant hand — CFC (yes/no)	210/26	N/A	0.066
Age	70.67/73.00	12.88	− 0.255⁎⁎
Education year	11.50/11.00	2.87	0.090
Scan time since stroke days	2.80/1.00	4.85	0.002
BCoS in days	12.32/11.00	8.23	− 0.250⁎⁎
Barthel index	14.66/16.00	5.14	0.333⁎⁎
Cognitive data			
Orientation (max = 8)	7.52/8	1.34	0.264⁎⁎
Auditory attention (max = 54)	43.17/50.00	14.08	0.414⁎⁎
Comprehension (max = 3)	2.85/3	0.39	0.294⁎⁎
Multi step object use (max = 12)	10.26/12.00	3.33	0.318⁎⁎
Gesture production (max = 12)	10.60/12.00	2.53	0.382⁎⁎
Gesture recognition (max = 6)	4.98/5.00	1.19	0.226⁎⁎
Meaningless imitation (max = 12)	9.76/10.00	2.58	0.489⁎⁎
Picture naming (max = 14)	10.74/12	3.42	0.413⁎⁎
Ego centric neglect (max = 20)	2.47/1	3.78	− 0.303⁎⁎
Complex figure copy (max = 47)	35.20/39.00	11.30	N/A

⁎⁎ *P* < 0.003 (after Bonferroni correction *p* < 0.05/16).

**Table 2:** PCA results on the re-scaled raw scores of the five praxis tests.

Tasks	PC1: high-level motor control	PC2: visuo-motor transformation in drawing	PC3: interacting with objects and planning	PC4	PC5
CFC	0.42	0.62	0.57	0.34	0.02
MOT	0.56	− 0.74	0.36	0.04	0.06
GP	0.42	0.16	− 0.45	− 0.10	0.76
GR	0.34	− 0.03	− 0.57	0.61	− 0.45
MI	0.46	0.21	− 0.13	− 0.71	− 0.47
Exp. var.	53%	18%	15%	8%	6%

Abbreviation: CFC: complex figure copy; MOT: multi-step object use; GP: gesture production; GR: gesture recognition; MI: meaningless imitation.

**Table 3:** VBM analysis based on the raw CFC scores after controlling for other correlated tests.

No control				Successively control for other cognitive tests			
Anatomy	BA			Praxis	Neglect	Attention	Picture naming
*Parietal lobe*							
R IPG	39	Cluster	1040**	934**	NA	NA	NA
Peak	4.01	4.29
x,y,z	[57,− 55,42]	[57,− 52 ,]

*Occipital lobe*							
R fusiform (extend to precun)	37	Cluster	448**	1810**	2698**	3185**	3062**
Peak	3.46	4.02	4.29	4.34	4.29
x,y,z	[32,− 55,− 6]	[32,− 55,− 6]	[32,− 55,− 6]	[32,− 55,− 6]	[32,− 55,− 6]
R lingual	18	Cluster	210	NA	%	NA	%
Peak	2.95		
x,y,z	[20,− 85,− 3]	[22,− 82,− 2]	[20,− 82,0]
L lingual	19	Cluster	1071**	1435**	1365**	1658**	1555**
Peak	3.81	4.00	4.03	4.32	4.19
x,y,z	[− 24,− 63,− 3]	[− 22,− 66,− 2]	[− 22,− 66,− 3]	[− 22,− 66,− 3]	[− 24,− 63,− 3]

*Subcortical*							
R Thalamus		Cluster	437**	380**	224**	165	305*
Peak	3.69	3.64	3.48	3.40	3.65
x,y,z	[21,− 15,10]	[21,− 12,12]	[21,− 12,12]	[21,− 12,12]	[21,− 13,10]
L thalamus		Cluster	270	783**	750**	437**	1363**
Peak	3.31	3.48	3.57	3.47	3.78
x,y,z	[− 21,− 16,12]	[− 18,− 10,9]	[− 18,− 10,9]	[− 18,− 10,9]	[− 18,− 10,9]

*Frontal*							
R aCG		Cluster	NA	380**	393**	994**	961**
Peak		3.30	3.39	3.80	3.74
x,y,z		[8,42,7]	[8,42,7]	[8,42,7]	[9,42,9]

*Cerebellum*							
R cerebellum — vermis		Cluster	NA	NA	178	NA	NA
Peak	3.22
x,y,z	[4,− 52,− 42]

FWE-correction at cluster level, *p < 0.05; **p < 0.01.

% right lingual cluster was part of the R FFG cluster reported in the row above it.

Abbreviation: R: right; L: left; IPG: inferior parietal gyrus; aCG: anterior cingulate gyrus; Cluster: Cluster size; Peak: Peak Z; x,y,z: x,y,z(mm).

**Table 4:** VBM analysis based on the three components indicated by the PCA.

Table a CP1 shared component (motor control)				
Anatomy	BA	Cluster size	Peak Z	x,y,z
R thalamus	NA	405**	3.36	22,− 33,16
L IPG	40	172	3.33	− 52,− 43,40
R MFG	6	398**	3.17	28,9,48
L postcentral G	4	272*	3.25	− 51,− 10,40

Table b CP2: CFC > MOT (visuo-motor transformation)				
Anatomy	BA	Cluster size	Peak Z	x,y,z

R MOG extending to R fusiform	19	1885**	3.98	30,− 81,3
L LG	19	1031**	3.75	− 26,− 63,− 2
L rolandic oper	48	433**	3.66	− 39,− 30,27

Table c CP3 CFC + MOT > gesture task (interacting with objects and planning)				
Anatomy	BA	Cluster size	Peak Z	x,y,z

L inf_frontal orb	47	260*	3.97	− 33,38,− 17
R LG	18	190	3.04	14,− 84,− 2
R precuneus		163	3.24	22,− 54,31
R fusiform	37	399**	3.25	42,− 39,− 15
Cerebellum	NA	298*	3.39	− 3,− 45,− 15

FWE-correction at cluster level, *p < 0.05; **p < 0.01.

Acronyms: R: right; L: left; BA: Brodmann area; IPG: inferior parietal gyrus; MFG: middle frontal gyrus; MOG: middle occipital gyrus; aCG: anterior cingulate gyrus; LG: lingual gyrus; Rolandic Oper: rolandic operculum; inf_frontal orb: inferior frontal orbital gyrus; Cluster: Cluster size; Peak: Peak Z; x,y,z: x,y,z(mm).

## Appendix A Supplementary data

Supplementary material Image 2
