# Supplementary material for: Lesion-symptom mapping of a complex figure copy task: A large-scale PCA study of the BCoS trial
Source: Neuroimage Clin. 2016 Apr 18;11:622–34. doi: 10.1016/j.nicl.2016.04.007 (PMC4857225; doi:10.1016/j.nicl.2016.04.007)
Supplement: Supplementary file 1 — Supplementary material [file mmc1.docx]

**Supp Table 1 Models comparison (log evidence)**

| ΔF | Model 1 | Model 2 | Model 3 | Model 4 | Model 5 |
| --- | --- | --- | --- | --- | --- |
| rFFG | | | | | |
| Model 1 |  | -387 | -653 | -285 | -459 |
| Model 2 | 387 |  | -266 | 102 | -63 |
| **Model 3** | **653** | **266** |  | **368** | **203** |
| Model 4 | 285 | -102 | -368 |  | -165 |
| Model 5 | 459 | 63 | -203 | 165 |  |
| rIPG | | | | | |
| Model 1 |  | 172 | -155 | 47 | -33 |
| Model 2 | -172 |  | -327 | -125 | -205 |
| **Model 3** | **155** | **327** |  | **202** | **122** |
| Model 4 | -47 | 125 | -202 |  | -80 |
| Model 5 | 33 | 205 | -122 | 80 |  |
| aCG |  |  |  |  |  |
| **Model 1** |  | **292** | **327** | **437** | **302** |
| Model 2 | -292 |  | 35 | 145 | 10 |
| Model 3 | -327 | -35 |  | 110 | -25 |
| Model 4 | -437 | -145 | -110 |  | -135 |
| Model 5 | -302 | -10 | 25 | 135 |  |

The table represents the log evidence difference between two models: ΔF = model in row – model in column.

Model 1：CFC control for orientation； Model 2：CFC control for orientation and the four praxis tasks; Model 3: CFC control for orientation, praxis and neglect tasks; Model 4: CFC control for orientation, praxis, neglect and attention tasks; Model 5: CFC control orientation for praxis, neglect, attention and picture naming tasks. The row representing the model that was most explanatory for each VOI is highlighted in BOLD.

We used a model comparison function in SPM (spm_vb_regionF.m) to compute the log evidence of each model for the specified ROI. The difference between the log evidence was used to infer on the best model. For each ROI we extracted the grey matter probability signal from the peak surrounded by a 6mm sphere and represented it using the first eigen variate. The results demonstrate that the best fitted model varies depending on the region selected and there is no one correct answer that fits all.

**Supp Table 2 SEM model comparison**

|  | AIC | | CAIC | CMIN/DF | GFI | | CFI | RMSEA |
| --- | --- | --- | --- | --- | --- | --- | --- | --- |
| CFC, Neglect and rIPC models comparison | | | | | | | | |
| Model 1 | 94.404 | | 253.081 | 1.441 | 0.977 | | 0.966 | 0.083 |
| Model 2 | 82.582 | | 239.258 | 1.258 | \| 0.989 \| \| --- \| | | 0.995 | 0.033 |
| Model 3 | 79.49 | | 240.642 | 0.832 | 0.933 | | 1 | 0 |
| Model 1 Model 2 Model 3  rIPG    CFC Neglect  rIPG    CFC Neglect  rIPG    CFC Neglect | | | | | | | | |
| aCG, CFC and Praxis tasks model comparison | | | | | | | | |
| Model 4 | | 114.063 | 248.357 | 9.011 | 0.951 | 0.894 | | 0.183 |
| Model 5 | | 77.658 | 225.381 | 3.886 | 0.988 | 0.981 | | 0.110 |
| Model 6 | | 71.200 | 223.400 | 1.600 | 0.997 | 0.997 | | 0.050 |
| Model 7 | | 69.127 | 203.421 | 1.521 | 0.991 | 0.993 | | 0.047 |
| Model 4 Model 5 Model 6 Model 7  aCG    CFC Praxis  aCG    CFC Praxis  aCG    CFC Praxis  aCG    CFC Praxis | | | | | | | | |

We used SEM to investigate in more details the relations between CFC, neglect and right IPC (Model 1-3) and the relation between CFC, aCG and the four praxis tasks (Model 4-7). The AIC values, which take into account the fitting accuracy and model complexity (number of parameters) was used to select the best fitting model. A smaller value of AIC represents a better model. Here we found that the correlation between CFC and rIPC are partially mediated via neglect, while the variability in CFC that cannot be accounted by praxis deficits was associated to aCG lesion. In the other hand, variability in CFC that cannot be accounted by praxis deficits was associated to aCG lesion.

The other value (CMIN/DF, CFI, GFI, RMSEA) displayed in the table represent the model fitting degree. Most of the parameters (CMIN/DF<3, GFI≥0.90,CFI>0.90, RMSEA<0.05) of Model 1-3, 6-7 fit well in our study.

**Supp Table 3 PCA result on the re-scaled raw scores of the five praxis tests, picture naming, neglect and sustained attention**

| Tasks | PC1 | PC2 | PC3 | PC4 | PC5 |
| --- | --- | --- | --- | --- | --- |
| **CFC** | -0.35 | -0.48 | -0.60 | -0.15 | 0.32 |
| **MOT** | -0.435 | 0.84 | -0.26 | -0.00 | 0.15 |
| **GP** | -0.36 | -0.15 | 0.26 | 0.20 | -0.21 |
| **GR** | -0.29 | -0.06 | 0.37 | 0.03 | -0.42 |
| **GMI** | -0.36 | -0.12 | -0.10 | 0.17 | -0.22 |
| **PN** | -0.40 | -0.14 | 0.15 | 0.59 | 0.31 |
| **NEG** | -0.11 | -0.03 | -0.47 | -0.08 | -0.70 |
| **SA** | -0.42 | -0.09 | 0.35 | -0.74 | 0.14 |
| **Exp. Var.** | **48%** | **11%** | **11%** | **8%** | **7%** |

Abbreviation: CFC: complex figure copy; MOT: multi-step object use; GP: gesture production; GR: gesture recognition; GMI: meaningless imitation PN：picture naming; NEG: neglect SA: sustained attention

Noted： 1.only the first 5 components were showed.

2. Since the higher score stand for the worse performance in neglect, we showed its opposite number here.

**Supp Table 4 VBM based on the three components teased apart from PCA(including picture naming, neglect and sustained attention).**

| **sTable 2a CP1 shared component (motor schemas, high-level motor control and proprioception processing)** | | | | | | | |  |
| --- | --- | --- | --- | --- | --- | --- | --- | --- |
| **Anatomy** | **BA** | **cluster size** | | **peak Z** | | **x,y,z {mm}** | |  |
| R superior Occipital | 19 | **296**** | | **3.29** | | **21 -78 28** | |  |
| R MFG | 8 | **975**** | | **3.96** | | **33 17 60** | |  |
| L postcentral G | 4 | **382**** | | **4.50** | | **-54 -15 52** | |  |
| **sTable 2b CP2: CFC > MOT (visual-motor transformation)** | | | | | | | |  |
| **Anatomy** | **BA** | **cluster size** | | **peak Z** | | **x,y,z {mm}** | |  |
| R MOG extending to  R fusiform | 19 | **1749**** | | **4.09** | | **30 -81 3** | |  |
| L LG | 47 | **665**** | | **3.63** | | **-30 -52 1** | |  |
| L Rolandic Oper | 48 | **307*** | | **3.42** | | **-39 -30 27** | |  |
| **sTable 2C CP3 CFC + MOT + NEG > Gesture task (interaction with objects, attention and planning)** | | | | | | | | |
| Anatomy | BA | | **cluster size** | | **peak Z** | | **x,y,z {mm}** | |
| R Inferior parietal extending to angular and supramarginal gyrus | 40 | | **2267*** | | **4.41** | | **63 -43 39** | |

FWE-correction at cluster level, *p < 0.05; **p < 0.01.

Abbreviation: R: right; L: left; BA: brodmann area; MFG: middle frontal gyrus; LG: lingual gyrus; Rolandic Oper: rolandic operculum; Cluster: Cluster size; Peak: Peak Z; x,y,z: x,y,z(mm)

**Supp figure 1 VBM analysis based on the first three components from PCA result**


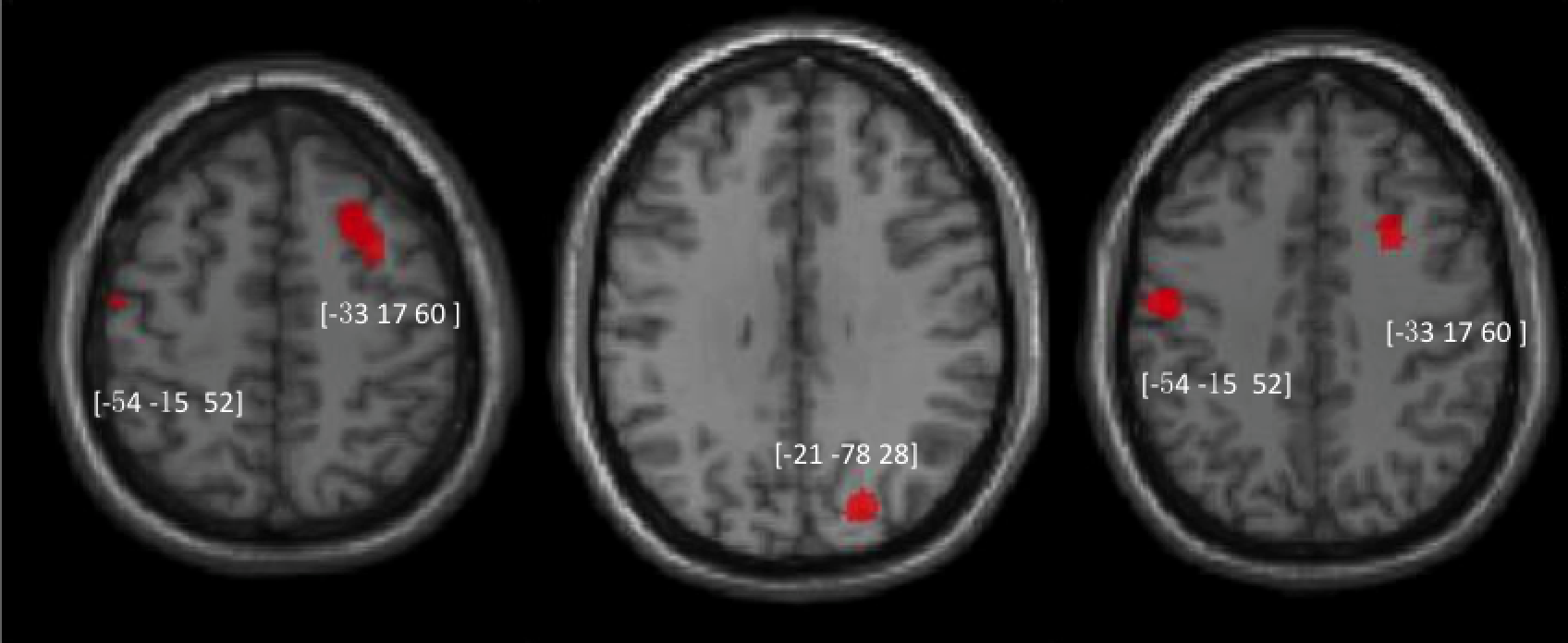


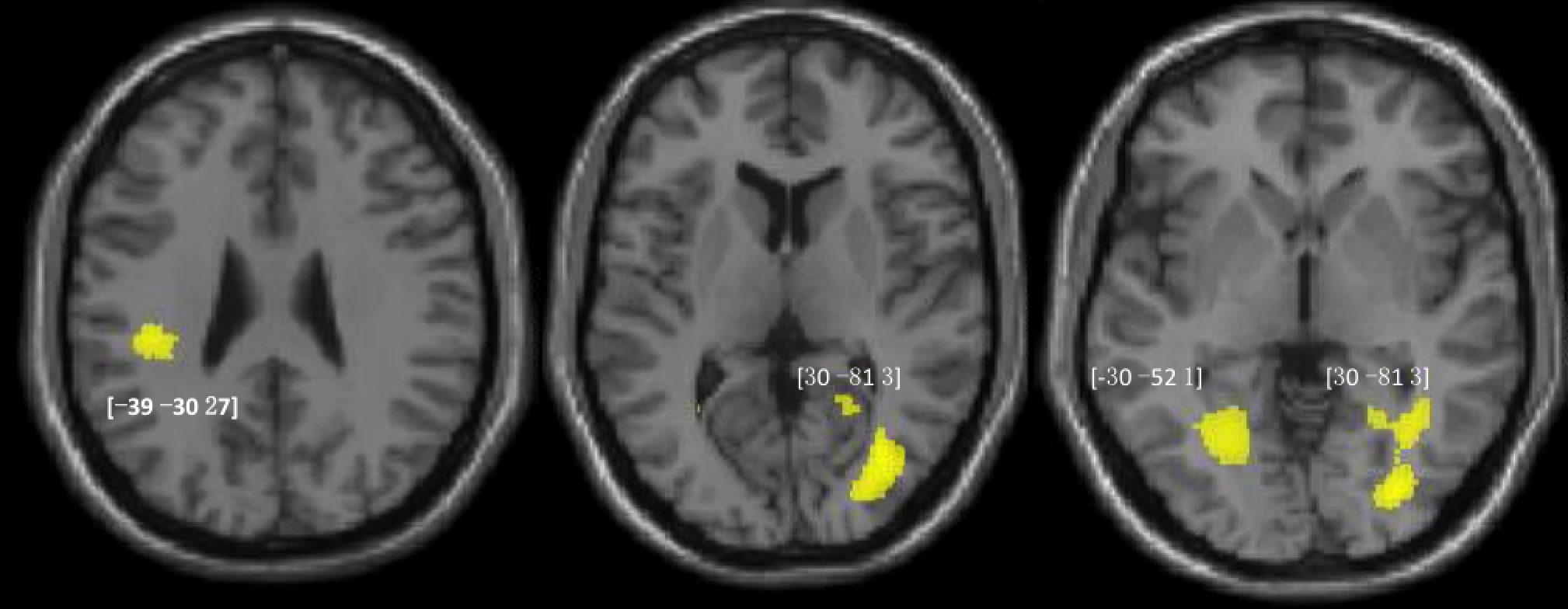

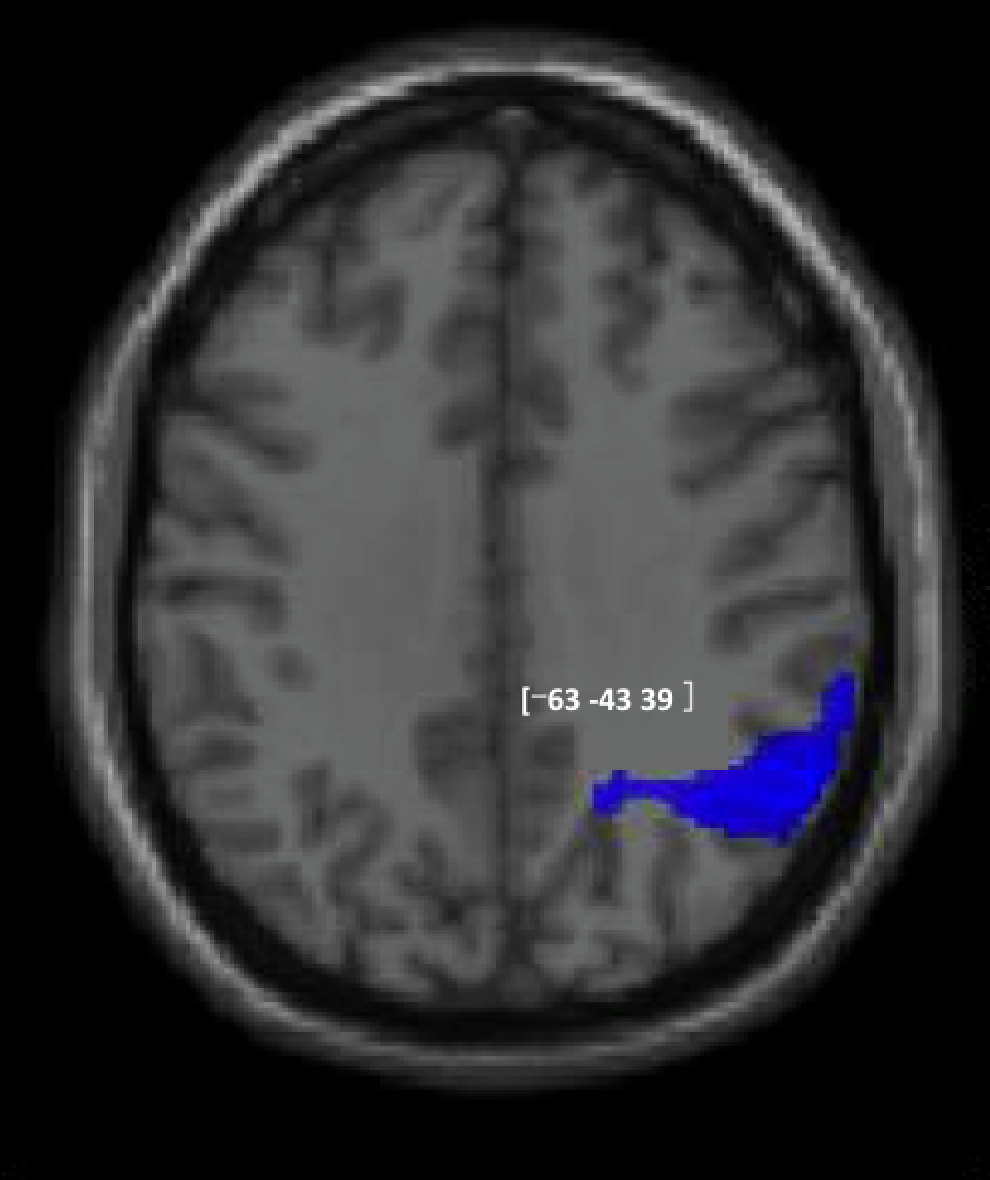


VBM results showing voxels corresponding to grey matter damage in (red) the first shared component, (yellow) the second component and (blue) the third component. The function-lesion maps are overlaid on axial T1-weighted MRI slices of the single subject canonical template provided by SPM. The numbers in brackets represent the peak of the clusters given in MNI coordinates.
